# An improved algorithm for model-based analysis of evoked skin conductance responses^☆^

**DOI:** 10.1016/j.biopsycho.2013.09.010

**Published:** 2013-12

**Authors:** Dominik R. Bach, Karl J. Friston, Raymond J. Dolan

**Affiliations:** a Wellcome Trust Centre for Neuroimaging, University College London, United Kingdom; b Berlin School of Mind and Brain, Humboldt University Berlin, Germany; c Zurich University Hospital for Psychiatry, Switzerland

**Keywords:** Skin conductance responses (SCR), Galvanic skin response (GSR), Electrodermal activity (EDA), General linear convolution model (GLM), Generative model, Model inversion

## Abstract

Model-based analysis of psychophysiological signals is more robust to noise – compared to standard approaches – and may furnish better predictors of psychological state, given a physiological signal. We have previously established the improved predictive validity of model-based analysis of evoked skin conductance responses to brief stimuli, relative to standard approaches. Here, we consider some technical aspects of the underlying generative model and demonstrate further improvements. Most importantly, harvesting between-subject variability in response shape can improve predictive validity, but only under constraints on plausible response forms. A further improvement is achieved by conditioning the physiological signal with high pass filtering. A general conclusion is that precise modelling of physiological time series does not markedly increase predictive validity; instead, it appears that a more constrained model and optimised data features provide better results, probably through a suppression of physiological fluctuation that is not caused by the experiment.

## Highlights

• We improve predictive validity of a general linear convolution method to analyse evoked SCR.
• A constrained individual response function provides highest predictive validity.
• This IRF is realised by a canonical SCRF together with its time derivative.
• A high pass filter of 0.05 Hz cut-off frequency is optimal for analysis.
• Non-linear models better reconstruct the observed time-series but have lower predictive validity.

## 1 Introduction

Recent interest in model-based analysis of skin conductance responses (SCR) (Bach & Friston, 2013) is – in part – motivated by the need to improve the temporal resolution of inference in rapid event-related paradigms (Barry, Feldmann, Gordon, Cocker, & Rennie, 1993). In model-based approaches, generative (forward) models specify how underlying physiological or psychological states generate observed data. Model inversion refers to estimating these (hidden) states from data. It turns out that inversion of probabilistic forward models has fundamental advantages, one of them being a propensity to suppress the effect of measurement noise (Bach, Flandin, Friston, & Dolan, 2009; Bach, Daunizeau, Friston, & Dolan, 2010; Bach, Friston, & Dolan, 2010; Bach, Daunizeau, Kuelzow, Friston, & Dolan, 2011). Statistical inference on the hidden states is generally more powerful than statistical comparisons of observed data because the models are more informed or constrained, leaving greater degrees of freedom in the data for efficient inference. Furthermore, the parameters of generative models provide a quantitative and explicit description of assumptions implicit in operational approaches (Bach & Friston, 2013), thus allowing for rigorous testing of those assumptions. Finally, model-based approaches afford quantitative rather than qualitative description of hidden, psychological processes.

Evoked skin conductance responses (eSCR) that follow a short (less than second) stimulus can be analysed with general linear convolution models – similar to the convolution models widely used in the analysis of functional magnetic resonance images (Friston, Jezzard, & Turner, 1994). In order to estimate the amplitude of evoked sympathetic nerve activity (SNA) from eSCR, we proposed such a convolution model (Bach et al., 2009). This model comprises two parts: a peripheral model incorporating a (standard linear time invariant) convolution operator, thought to be implemented by the sudomotor nerve terminal and sweat gland; and a linear neural model assuming infinitely short neural bursts immediately after each stimulus. We have shown that time invariance assumptions for the peripheral system are largely met (Bach, Flandin, Friston, & Dolan, 2010), while non-linearities in the peripheral system may occur but can easily be modelled within this framework – see (Bach & Friston, 2013) for a discussion. The model is highly regularised by placing informative constraints on the form or shape of the convolution kernel which models the peripheral response function (RF). This enables one to estimate the mean evoked SNA amplitude for each condition of an experimental design – even when observed eSCR overlap in time. This model was designed to optimise the predictive power of the estimates, rather than to precisely reconstruct the observed time series. Indeed, when subjects observe negative-arousing or neutral pictures, picture category can be better predicted from SNA estimates than from SCR peaks, an observation that speaks to its predictive validity (Bach et al., 2009).

Model-based eSCR analysis, based on probabilistic inversion of a general linear convolution models, is thus a potentially powerful method. As with any method, however, the practical implementation makes certain technical assumptions that go beyond the known biophysical properties of the system. Three points deserve particular attention:

Firstly, the peripheral response model uses a canonical skin conductance response function (SCRF) for all experiments and individuals. Such a stereotypical response function is a strong biophysical assumption and unsupported by observation. Indeed in our own validation experiments, we observed large inter-individual variability, accounting for up to 20% of overall response variability (Bach, Flandin, et al., 2010). Therefore, we added orthogonalised Taylor expansions to the SCRF to account for differences between individuals and conditions, thus improving model fit (Bach et al., 2009). Effectively, this enables the model to fit a subject specific RF in terms of a linear mixture of basis functions of peristimulus time, where the basis set is generated by the Taylor expansion. However, because the additional basis functions are orthogonalised to the SCRF, they do not affect the estimation of the parameter controlling the amplitude of the SCRF (Calhoun, Stevens, Pearlson, & Kiehl, 2004; Hopfinger, Buchel, Holmes, & Friston, 2000). Yet, this parameter is taken to estimate the SNA. This means that additional basis functions improve data fit at the within subject level but not comparisons of SNA at the between subject level. Hence, one might ask whether modelling an individual response function (IRF) – rather than a canonical skin conductance response function (SCRF) – improves predictive validity.

There are several ways to model subject specific IRF. First, the SCRF together with the remaining basis functions can be used to estimate a subject and condition specific IRF. That is, we can reconstruct the estimated eSCR, measure the peak amplitude (over peristimulus time) and use this as an estimate of SNA amplitude, instead of the canonical parameter estimate. Other regularised basis sets also provide models of IRFs. An uninformed finite impulse response (FIR) model was proposed in Bach et al. (2009) due to its popularity in fMRI research. A cosine set also used in fMRI analysis serves the same purpose. These basis sets typically have a larger number of basis functions than basis sets built upon truncated Taylor expansions. This means that although they are more flexible, they require greater numbers of parameters to be estimated. In all these approaches, separate IRFs are estimated for each condition within one individual. A more informed approach is to assume the form of the subject specific response function is the same for all experimental conditions. We have implemented this constraint by extracting data from all conditions and fitting a response function to the first principal component of the data. We will refer to this response function as the subject-specific response function (SRF).

A second issue is that skin conductance time series comprise both phasic responses and a slowly drifting tonic component. This drift is why many analysis schemes high pass filter the signal (Boucsein, 2012), including contemporary model-based approaches (Benedek & Kaernbach, 2010a, 2010b). This renders the phasic responses finite and removes slow signal drifts which are difficult to model. In our implementation, we used a bidirectional first-order Butterworth filter with time constant of 10 s (corresponding to a cut off frequency of 0.0159 Hz) (Boucsein, 2012). A bidirectional filter was chosen as it retains peak latencies. Because this filter can slightly distort the shape of the signal, the regressors of the general linear convolution model are subjected to the same filter. The choice of the filter frequency is based on prior experience but not on theoretical considerations. Therefore, one may ask whether there is an optimal filter that provides the best data features for modelling. Generally speaking – when modelling biological time series – data features that cannot be produced by a plausible forward model are probably measurement noise or the product of hidden processes not included explicitly in the model. This usually means they can be discarded with impunity, thereby increasing the signal-to-noise ratio (SNR) of the pre-processed data. Data conditioning is then, effectively, a part of model inversion. The question here is whether there is an optimal high pass filtering of skin conductance timeseries that increases signal-to-noise. In case of a signal with precisely known RF, the matched filter theorem provides a way of theoretically deriving a filter that maximises the SNR. In our case, the true RF is not precisely known, and also varies between individuals, such that we sought to empirically determine the filter characteristics that maximise predictive validity of SN estimates.

Finally, a linear neuronal model makes the strong assumption that SNA evoked by a short stimulus occurs with constant latency. We have previously shown that under this assumption there is no evidence for time-varying responses in the peripheral system (Bach, Flandin, et al., 2010). Here, we revisit this assumption and investigate whether modelling variations in neuronal latency improves predictive validity, under the assumption of an invariant peripheral response. Hence, we compare linear and non-linear models. Two particular non-linear models are considered. First, we used our previous approach that uses Dynamic Causal Modelling (DCM) (Bach, Daunizeau, et al., 2010) to obtain estimates of SNA amplitude per trial by letting response amplitude and onset vary on a trial-by-trial basis. Note that the neural model here is still informed insofar as it specifies a certain response window. Some authors propose uninformed neural models; in other words, they assume that SNA can occur any time, but to only use SNA during post-stimulus time windows for analysis (Benedek & Kaernbach, 2010a, 2010b). We sought to emulate this approach using DCM for spontaneous fluctuations (Bach et al., 2011). Both approaches yield a trial-by-trial estimate of SNA amplitude, which was averaged across experimental conditions for comparison with other approaches.

In some circumstances – e.g. to use neural response estimates as explanatory variables for analysis of independent experimental data, such as fMRI, trial-by-trial estimates of SN amplitudes may be required. Here, we sought to establish whether linear models are sufficient for this purpose or whether the iterative procedures required for non-linear models inherent in DCM are justified.

We have previously discussed how to benchmark methods that estimate hidden variables from observed data (Bach & Friston, 2013). One way is by making certain assumptions about what causes the hidden variable to change. A consensus assumption in the psychophysiology literature is that emotionally arousing events increase sympathetic arousal, as engendered by negative and positive arousing images. This has been demonstrated using operational approaches (Amrhein, Muhlberger, Pauli, & Wiedemann, 2004; Greenwald & Lang, 1989; Johnsen, Thayer, & Hugdahl, 1995; Winton, Putnam, & Krauss, 1984). Here, we assume that negative and positive arousing images would elicit greater sympathetic arousal than neutral non-arousing images, and evaluated different models in terms of their ability to distinguish between image types, using just the observed SCR.

In summary, we evaluated our method empirically, by comparing the predictive validity of different generative models. In a first step, we compared a canonical response function against various forms of an individualised response functions (IRF). Taking the best model from this step, we then compared various filter settings, non-linear methods, and the efficiency of trial-by-trial estimates. These comparisons used two independent, previously unpublished data sets.

## 2 Methods

### 2.1 Datasets

We analysed two datasets, both acquired during a study that examined the influence of distracting background noise on the perception of emotionally arousing images. There was no interaction of acoustic distractors with differential SCR in either experiment. The main effects of auditory stimulation will be reported elsewhere. For the present analysis, all data were collapsed across the auditory stimulation factor. Participants for both experiments were recruited from UCL students and in the general population via an online recruitment system. Both experiments were approved by the Joint UCL/UCLH Committees on the Ethics of Human Research.

The first experiment investigated SCR in response to negative-arousing and neutral pictures. 60 healthy individuals (30 male, 30 female; age: *M* = 23.7; SD = 4.7 years) participated in the first experiment. Participants watched, in randomised order, the 45 least arousing neutral (valence within 1 standard deviation around the mean) and 45 most arousing aversive pictures (valence lower than 1 standard deviation below the mean) from the International Affective Picture Set [IAPS] (Lang, Bradley, & Cuthbert, 2005) for 1 s each, with an inter stimulus (ISI) interval randomly determined as 7.65 s, 9 s, or 10.35 s. Participants were instructed to press the cursor up or down key on a computer keyboard to indicate whether they liked the picture or not. This response served to increase SCR. The experiment was divided into three blocks with 45 s breaks in between.

The second experiment was similar to the first, with an additional third condition of positive arousing images. 40 healthy individuals (20 male, 20 female; age: *M* = 21.9; SD = 3.8 years) watched the 16 least arousing neutral, most arousing aversive and most arousing positive images (defined analogous to experiment 1, and excluding explicit nude images) from the IAPS, for 1 s each, in randomised order, and in one single block. ISI was 4.4 s. Responses were the same as in experiment 1. Lists of images, used in both experiments, are available from the authors.

### 2.2 SCR recordings and preprocessing

We recorded skin conductance on thenar/hypothenar surface of the non-dominant hand using 8 mm Ag/AgCl cup electrodes (EL258, Biopac Systems, Goleta CA, USA) and 0.5%-NaCl electrode paste (GEL101; Biopac Systems), using a custom-build constant voltage coupler (2.5 V). The output of the coupler was converted into an optical pulse frequency with an offset (i.e. minimum sampling rate) of 100 Hz and a factor of 31 Hz/μS; the converter was designed for a maximum conductance of 50 μS, corresponding to 1650 Hz pulse frequency. The optical signal was converted to voltage pulses and recorded (Micro1401/Spike 2, Cambridge Electronic Design, Cambridge, UK). SCR data were filtered with a 1st order Butterworth filter. A bidirectional filter with low pass cut off frequency of 5 Hz was used, and a bidirectional high pass filter with cut off frequency of 0.0159 Hz for step 1. High pass filter settings were variable for steps 2–4. Data were then down sampled to 10 Hz.

### 2.3 Inversion schemes and models

In steps 1 and 2, the data were analysed with a general linear convolution model as proposed in (Bach et al., 2009). Additional models were compared in step 3–4, see below. For each experimental condition, event onsets were modelled as Dirac delta functions and convolved with a single RF or basis set as described below. The resulting design matrix of the general linear model contained one regressor for each condition and function in the basis set. For basis sets containing more than one basis function, the amplitude of the underlying SNA was estimated as the peak of largest modulus, of the reconstructed SCR from all basis functions in the basis set. To ensure that these findings could be generalised from the detection of categorical condition-specific differences to continuous variables, we additionally analysed a model for experiment 1 in which picture onset was modelled by a single stimulus function for both conditions, and picture type and linear habituation effects were modelled as parametric modulators. This models the linear component of the (possibly non-linear) habituation of SNA amplitude over the course of an experiment.

### 2.4 Step 1: response functions

The basis sets we assessed are listed below. The number of basis functions (bf) and of free parameters (*k*) for a model containing *j* conditions and *N* trials are noted in brackets for each basis set. For basis sets containing more than one basis function, the ensuing regressors were orthonogalised using a serial Gram-Schmidt procedure:

(0) Peak scoring: several peak scoring methods were computed to allow comparison with the literature. We followed the recommendations of the Society for Psychophysiological Research (SPR) (Boucsein et al., 2012) to identify an SCR according to the onset latency, identified by the point of maximum deflection, together with the rise-time (i.e. onset-peak latency) of the subsequent peak. Because there is no community consensus on the optimal duration of the onset latency window, we used a window of 1–4 s (Boucsein, 2012; Dawson & Filion, 2007; Edelberg, 1972) and a window of 1–3 s (Barry, 1987; Boucsein, 2012; Dawson & Filion, 2007), and a post-onset peak window of 0.5–5 s (Boucsein, 2012). Onset SCR values were subtracted from peak SCR values. Non-responses were scored zero. For both windows, we calculated SCR amplitude by omitting responses below 0.01 μS before averaging, and SCR magnitude by averaging all responses including zero responses. Further, we used a simpler algorithm and subtracted the mean of a 1 s pre-stimulus from the highest value in a 1–4 s post-stimulus response window.

(1) SCRF: the standard SCRF (bf = 1, *k* = *j*) – this is the reference model for steps 1 and 3.
(2) SCRF/time derivative: the standard SCRF with time derivative (bf = 2, *k* = 2*j*) – this is the reference model for step 2.
(3) SCRF/time and dispersion derivative: the standard SCRF with time and dispersion derivative (bf = 3, *k* = 3*j*).
(4) FIR 15 s: an uninformed finite impulse response basis set with 15 post-stimulus time bins of 1 s duration (bf = 15, *k* = 15*j*).
(5) FIR 30 s: an uninformed finite impulse response basis set with 30 post-stimulus time bins of 1 s duration (bf = 30, *k* = 30*j*).
(6) Cosine 4th order: A 4th order cosine set of 60 s duration (bf = 9, k = 9j)
(7) Cosine 8th order: A 8th order cosine set of 60 s duration (bf = 17, *k* = 17*j*).
(8) SRF: a model with subject-specific RF. This was evaluated by extracting the 8.65 s after each stimulus onset (corresponding to the duration of the shortest stimulus onset asynchrony [SOA]), calculating a principal component analysis over epochs, and extracting the first component. A response function was then fitted to the first principal component with a 2nd order ordinary differential equation, using a variational Bayes inversion scheme as described in (Bach, Daunizeau, et al., 2010). This form of model was considered for experiment 1 – in experiment 2, the minimum SOA was too short to estimate a robust SRF (bf = 1, *k* = 3 + *j*).

### 2.5 Step 2: filter settings

In the second step, we compared different filter settings for the best method from step 1 (SCRF with time derivatives) which we define as reference method for step 2. Uni- and bidirectional 1st order Butterworth high pass filters were compared with cut-off frequencies of 0.005 Hz, 0.1 Hz, 0.0159 Hz (the current default), and from 0.02 Hz to 0.10 Hz in 0.005 Hz steps. Bidirectional filtering, as implemented in the Matlab function filtfilt.m, filters the time series twice, in the forward and backward direction. To keep filter order precisely the same, we filtered twice for unidirectional filtering, but both times in forward direction. To ensure the robustness of these findings, the evaluation of different filtering was also performed using a simple SCRF without derivatives.

### 2.6 Step 3: non-linear models

(1) Informed DCM: In the first non-linear model, an SN burst was assumed to occur within 2000 ms after stimulus onset – this differs from the linear model where it was assumed to occur immediately after stimulus onset. The onset, duration and amplitude of each SN burst was estimated using DCM as described in (Bach, Daunizeau, et al., 2010) (*k* = 3*N*).
(2) Uninformed DCM: In a second non-linear model, SN bursts were assumed to occur anywhere within the experiment (with fixed dispersion to constrain the number of free parameters) – analogous to our DCM for spontaneous fluctuations (Bach et al., 2011). Due to the large numbers of parameters, the model could not be inverted, so a 60 s epoch was inverted every 30 s. From the overlapping epochs, the middle 30 s were analysed. Each estimated SN response that caused an SCR that fell into a 1–4 s post stimulus time window was extracted, and the largest response in each window was retained. This model is entirely uninformed about the experiment, in line with other contemporary model-based approaches (Benedek & Kaernbach, 2010a, 2010b) (*k* = 60*e*, where *e* is the number of inverted data epochs).

Both non-linear models were compared to the reference model from step 1.

### 2.7 Step 4: trial-by-trial estimates

All analyses described thus far estimated SNA amplitude under the assumption that the responses were the same for each trial in a condition. In some circumstances one might want to estimate trial by trial SNA. To evaluate the validity of trial by trial estimates, we assumed that arousal ratings from the validation sample of the IAPS (Lang et al., 2005) and habituation were the principal causes of trial by trial variations. We therefore quantified the proportion of variance in trial-by-trial estimates explained by these variables. We analysed single trials from experiment 1 and computed a GLM as explained above, with one regressor per trial, using either the SCRF or SCRF with time derivative, and a high-pass filter with cut off frequency of 0.05 Hz. For comparison, a DCM was inverted where SCR were modelled as evoked responses with constant latency. This is a linear neural model but using a non-linear, iterative inversion scheme. Finally, we used trial-by-trial SNA from the informed DCM from step 3. These four models were compared against each other.

### 2.8 Model comparison

The different models and data features were compared in terms of their predictive validity; i.e., their ability to predict a stimulus class from estimated SNA, for a particular experimental contrast. The contrast of interest for experiment 1 was the difference between neutral and negative-arousing pictures. For experiment 2, we used the contrasts neutral vs. negative-arousing and neutral vs. positive-arousing. To assess predictive validity, we used a general linear model with the contrast of interest as the response variable, and the estimated SN activity as predictor. The design matrix included subject effects. The residual sum of squares RSS was converted to a negative log likelihood value LL, such that smaller LL values indicate a higher predictive validity using the following relation

(1) $$\text{LL}=n\log\left(\frac{1}{n}\text{RSS}\right)$$

where *n* is the number of observations. This disregards model complexity, which was the same for all analyses. We report log evidence differences or log Bayes Factors (LBF) – the difference in log likelihood between each model and a reference model, for each step in model comparison. Negative LBF values indicate a model fit that is better than the reference model. An LBF difference larger than 3 is often considered decisive as it corresponds to a *p*-value of 0.05.

We further compared how well the models fit the observed SCR time series given the optimised parameters. This was done for the sake of completeness, although this measure of model performance should be interpreted with caution because (within subject) measures of accuracy, such as this, do not reflect predictive validity at between subject level. We converted the residual sum of squares RSS from the fitted time series into a negative log likelihood value LL using Eq. (1) which was then corrected for complexity to give the Akaike information criterion (AIC) by the relation

(2) AIC=LL+2k

where *k* is the number of free parameters in the model. AIC is an approximation to Bayesian model evidence (Penny et al., 2010). For step 4, we converted the relative residual variance into negative log likelihood by Eq. (1), and summed up the likelihood terms across participants. For model fit, we report Log Bayes Factors (LBF) – the difference in AIC between each model and a reference model.

## 3 Results

The stimuli had a clear effect on estimated SNA as demonstrated by all analyses. For the reference (standard SCRF) method, negative-arousing images elicited higher SNA than neutral images in experiment 1 (*t*(59) = 4.70; *p* < .00001), and in experiment 2 (*t*(38) = 4.68; *p* < .00001). The difference in activity between positive and neutral images (*t*(38) = 1.44; *p* = 0.16) failed to reach significance in the reference method but was significant in other model-based methods.

### 3.1 Step 1: optimising the response function (RF)

**Fig. 1:**
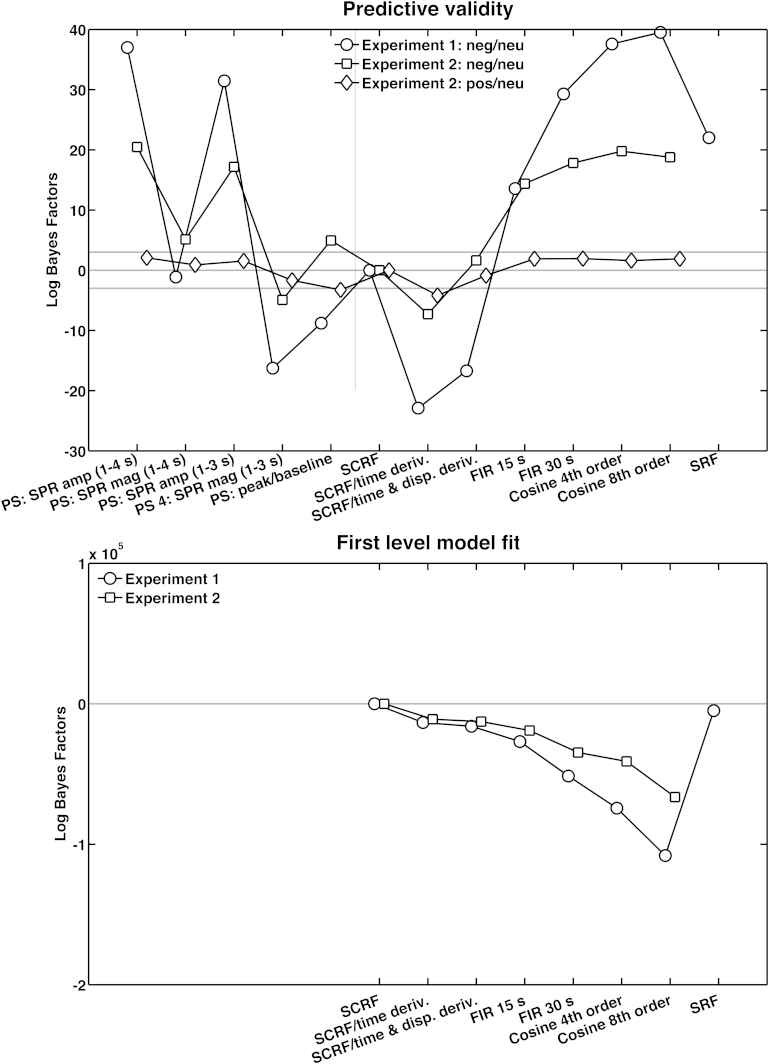
Step 1: comparison of a linear model for evoked SCR, using different response functions as explained in Section 2. Lower Log Bayes Factors (LBF) indicate higher model evidence for the target model. *Upper panel*: predictive validity; i.e., ability of estimated SN amplitudes to predict a known sympathetic state, for three contrasts from two experiments, expressed in LBF as negative log likelihood difference between the model in question and a reference model. Several peak scoring methods (PS) are added for illustrative purposes as null models (left of the dashed line). *Lower panel*: Model Log evidence of the within-subject model, expressed as difference in AIC between the target model and our benchmark model, summed over participants. *Abbreviations*: PS: SPR (1–4 s) amp – peak scoring amplitude according to the SPR recommendations, using a 1–4 s post-stimulus onset window; PS: SPR (1–4 s) mag – peak scoring magnitude according to the SPR recommendations, using a 1–4 s post-stimulus onset window; PS: SPR (1–3 s) amp – peak scoring amplitude according to the SPR recommendations, using a 1–3 s post-stimulus onset window; PS: SPR (1–4 s) mag – peak scoring magnitude according to the SPR recommendations, using a 1–4 s post-stimulus onset window; PS: peak/baseline – peak scoring magnitude, substracting a 1 s pre-stimulus baseline from the maximum value within a 1–4 s post-stimulus window; SCRF – skin conductance response function (benchmark method); SCRF/time deriv. – skin conductance response function with time derivative; SCRF/time and disp deriv. – skin conductance response function with time and dispersion derivative; FIR 15 s – uninformed finite impulse response function with 15 timebins of 1 s duration; FIR 30 s – uninformed finite impulse response function with 30 timebins of 1 s duration; Cosine nth order – cosine basis set of nth order; SRF – subject-specific response function.

We compared all models against our reference model with SCRF alone, and added a peak scoring approach for comparison with the literature. Fig. 1a shows that, for all contrasts and among linear models, SNA estimates from a model including the SCRF and its time derivative best predicted the experimental manipulation; i.e., they had the lowest Log Bayes Factor (LBF). The LBF difference between this and the reference method was decisive for all three contrasts. Estimates from linear models with more degrees of freedom had equal or lower predictive validity (i.e. higher LBF) than the reference. At the same time, the best linear model was better than any of the peak scoring methods.

In order to assess whether this result was due to modelling of dichotomous contrasts between experimental conditions or whether it would generalise to continuous predictors, we modelled condition and habituation in experiment 1 as parametric modulators. Again, an SCRF with time derivative model had best predictive validity, both for the effect condition and the continuous predictor, the linear habituation term (data not shown).

In terms of within-subject model fit, we found a different picture (Fig. 1). More complex models had a massively improved model fit, despite penalising model complexity, while the predictive validity of these methods tended to be worse. This might indicate that the more complex models over-fit noise variance not induced by the experimental manipulation.

In summary, we conclude that a linear model using SCRF and time derivative provides the approach with the greatest predictive validity (among the ones considered here). This appears to be the case over the two experiments with different experimental contrasts. Hence, we used this model for the next evaluation step.

### 3.2 Step 2: optimising the filter

**Fig. 2:**
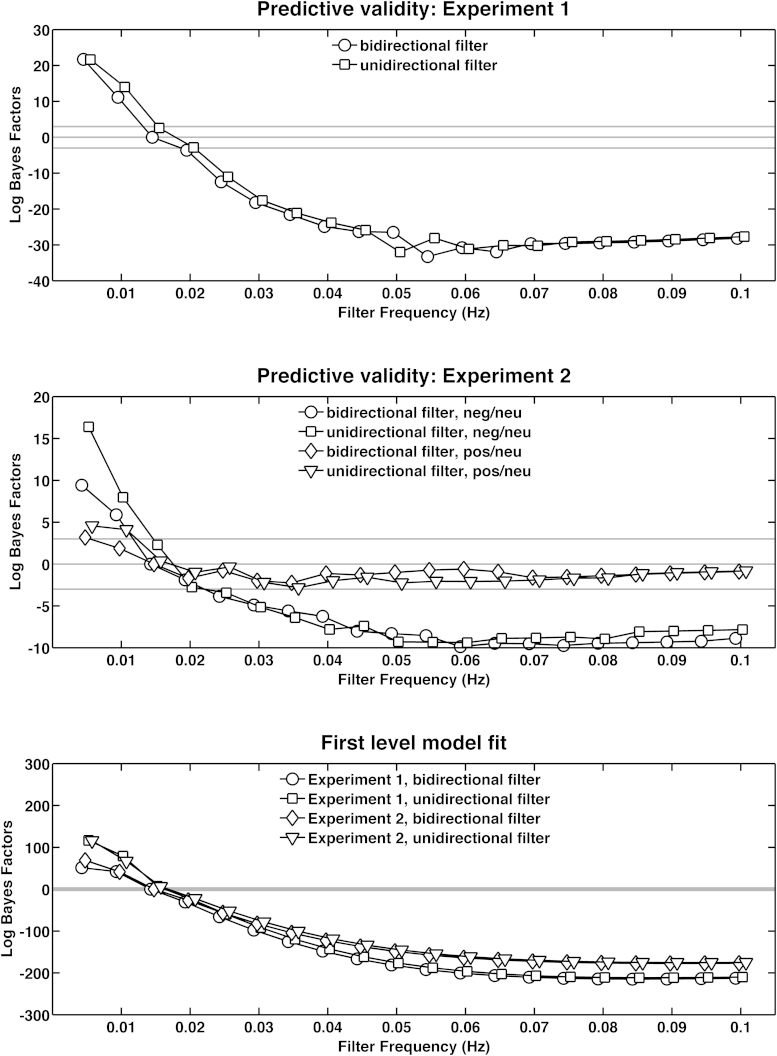
Step 2: comparison of different unidirectional and bidirectional high pass filters, applied to the data before model inversion. Lower Log Bayes Factors indicate higher target model evidence. All Log Bayes Factors are with respect to our current standard filter, a bidirectional first order Butterworth filter with cut off frequency of 0.0159 Hz.

All filter settings were referenced against the winning method from step 1, which estimates SN amplitude from a reconstructed response function, using a general linear convolution model with a canonical SCRF and time derivative, at standard high pass filter settings – a bidirectional first order Butterworth filter with cut-off frequency of 0.0159 Hz. In Fig. 2, this model hence has a LBF of zero. We varied filter cut-off frequency from 0.005 to 0.1 Hz, and employed a unidirectional and bidirectional filter.

Fig. 2 shows that increasingly higher filter frequencies produced SN response estimates with better predictive validity, up to about 0.05–0.06 Hz for the contrast negative > neutral in both experiments, and up to about 0.035 Hz for the contrast positive > neutral from experiment 2. Beyond this frequency, predictive validity did not change much, but tended to get slightly smaller towards higher frequency cut-offs. There was no clear advantage for either uni- or bidirectional filtering. Unidirectional filters tended to be slightly better for higher frequency and vice versa for lower frequency. Note that both uni- and bidirectional filtering applied the filter twice, but in different directions. The same pattern of results was found when the SCRF was used without derivatives (data not shown), indicating that the influence of filter is not due to peculiarities of the response function.

As in step 2, the effect of the filter upon predictive validity was not precisely reflected in first-level model fit. Model fit asymptotically increased with higher filter cut-off frequencies – as one might expect when degrees of freedom are removed from the data (making it easier to fit).

To summarise, higher filter cut-off frequencies produced better predictive validity. However, across the different contrasts, the precise choice of filter frequency had no decisive and unambiguous effect on predictive validity between a frequency of 0.035 and 0.1 Hz.

### 3.3 Step 3

In the third step, we compared linear and non-linear models which model more degrees of freedom in underlying SNA latency. The DCM for event-related responses allows variability in the neural response timing on a trial-by-trial basis and might thus be able to estimate SN amplitude even if neural latencies fluctuate. DCM for spontaneous fluctuations makes even less assumptions about the neural response and can capture different shapes of neural responses, e.g. mono- or biphasic.

**Fig. 3:**
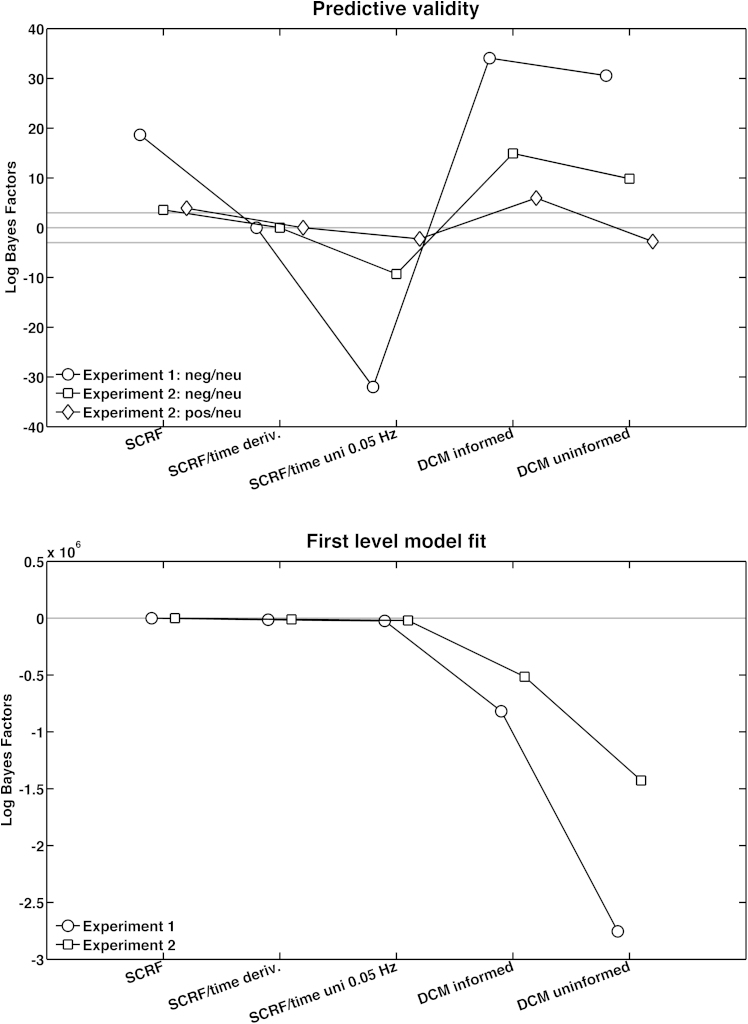
Step 3: comparison of several linear with non-linear models for evoked responses. Lower Log Bayes Factors indicate higher target model evidence.

Fig. 3 shows that the DCM for event-related responses – labelled informed because it is informed about the experimental timing – had decisively lower predictive validity than any linear model for all three contrasts. The uninformed DCM had lower predictive validity than linear models for the contrast neutral > negative, across both experiments. For the contrast positive > neutral, it had approximately the same predictive validity as the best linear model. As in the previous steps, a better model fit under more complex models with more degrees of freedom was not reflected in predictive validity.

### 3.4 Step 4

The single-trial GLM (with two different RFs) and a DCM for evoked responses performed almost equally well: the mean explained variance proportion across participants was (mean ± standard deviation) 0.15 ± 0.13, 0.16 ± 0.14, 0.17 ± 0.13, respectively, while the non-linear DCM for event-related responses performed slightly worse, with an explained variance proportion of 0.12 ± 0.09. Model evidence was decisively higher for the iterative DCM for evoked responses than for all other approaches.

## 4 Discussion

Model-based methods for analysis of SCR responses estimate underlying SNA and are potentially important for non-invasive measurements of autonomic responses. Here, we sought to validate and improve a method based upon linear and nonlinear convolution models – with three results.

First, as general validation of linear models, the best linear method had as good, or better, predictive validity than various peak-scoring approaches based directly on observed data, or than all non-linear methods. At the same time, first-level model fit was unambiguously higher for models with higher complexity, in particular for non-linear models. This demonstrates that the tight constraints imposed by linear models preclude overfitting, and to help recover the underlying hidden cause more efficiently.

Second, the original linear method was improved by allowing for between-subject variability in the form of peripheral responses. The best predictive validity was achieved with a model that used a standard canonical SCRF across subjects, together with its temporal derivative, to estimate an individual and condition specific RF. It is noteworthy that the approach was also best when the binary variable (experimental condition) was encoded as parametric modulator, or when predicting a continuous variable (linear response habituation) encoded as parametric modulator. This approach of complementing the SCRF with Taylor expansions was already implicit in the original model proposed in (Bach et al., 2009; Bach, Flandin, et al., 2010); but in the original method it was only used to improve within-subject model fit, not to estimate SNA amplitude. Here, we extend this approach and propose this as a future standard for analysis of eSCR. Note that this remains a constrained or informed way of estimating IRFs. Other approaches with even slightly higher complexity did not improve predictive validity. In a nutshell, the winning method uses the same approach that has been proven most sensitive in fMRI analysis (Hopfinger et al., 2000). It is interesting to note that a more constrained approach, namely the estimation of a subject-specific response function (SRF) from the data without allowing for condition differences, proved less powerful. One reason might be that the particular algorithm used only a short post-stimulus time window for estimation of the SRF, different from all other approaches; this might cause inaccuracy in the estimated SRF.

Thirdly, predictive validity could be improved by optimising high-pass filtering. A filter is commonly applied in SCR analysis to remove low-frequency drifts and condition the data. Filter choice has been based on convention or upon considerations about the response frequency spectrum (Boucsein, 2012). Here, we used an empirical approach to approximate the predictive validity of a linear method with different filter settings. We found an optimal filtering between 0.035 and 0.06 Hz with no decisive impact of filter frequency on predictive validity over this frequency range. As an intermediate choice, we suggest a 0.05 Hz unidirectional 1st order Butterworth filter as appropriate, in the context of general linear convolution modelling for eSCR.

We note that for a contrast between positive-arousing and neutral images, some aspects of the model comparison were less pronounced than for the contrasts between negative-arousing and neutral images. In particular, the precise choise of optimal filter frequency was less clear for the latter contrast. Furthermore, a non-linear model-based method – that was not informed about the experimental design – had as high a predictive validity as the best linear model. This unexpected finding differed from findings from a contrast of negative-arousing and neutral images. A possible reason is that all other methods assume a monophasic response – if responses are biphasic, these methods will be suboptimal. However, the DCM for event-related responses can easily be extended to biphasic responses as demonstrated in a previous report (Bach, Daunizeau, et al., 2010). We hope to look at this possibility in a future experiment that is optimised to assess response shape. However, no method performed better for the contrast of positive-arousing and neutral images than the best linear model, and the overall pattern of results was the same for all contrasts. The fact that results are slightly less clear for this particular contrast might also reflect that the assumption of a difference in sympathetic arousal between positive and neutral images is not as unambiguously supported as for negative and neutral images (where all methods captured this difference, to a variable extent): some studies using operational approaches have suggested that sympathetic arousal in response to positive imagery might be restricted to its particular contents (e.g. explicit pictures which were not used in the present study) (Bernat, Patrick, Benning, & Tellegen, 2006).

Note we did not test the ability of the various methods to detect responses within one condition, as in a one-sample *t*-test on estimated SNA amplitudes for one condition. This is because such a comparison is biased and will favour methods that have skewed null distributions such as peak-scoring, model-based methods using reconstructed response functions, or non-linear models where the neural response is specified to be positive.

Finally, linear models are better able to recover SN amplitudes on a trial-by-trial basis than a non-linear DCM, although a linear, but iterative DCM for evoked responses was even better, as indicated by higher proportion of explained variance in the amplitude estimates. Note that generally the proportion of explained variance in single-trial SNA estimates is relatively low (around 0.15), reflecting large within-condition variability.

In summary, we have further validated a previously proposed linear convolution model approach to estimating SNA evoked by short arousing events. We were able to improve the scheme by augmenting the peripheral response function basis set and the filtering settings. The improved algorithm will be available as standard method in future releases of the software SCRalyze, available at http://scralyze.sourceforge.net.
